# The Grapefruit Effect: Interaction between Cytochrome P450 and Coumarin Food Components, Bergamottin, Fraxidin and Osthole. X-ray Crystal Structure and DFT Studies

**DOI:** 10.3390/molecules25143158

**Published:** 2020-07-10

**Authors:** Miriam Rossi, Sandjida Aktar, Marissa Davis, Emily Hefter Feuss, Samara Roman-Holba, Kelly Wen, Christopher Gahn, Francesco Caruso

**Affiliations:** 1 Chemistry Department, Vassar College, Poughkeepsie, NY 12604, USA; sandjidaaktar123@gmail.com (S.A.); rissacldavis@gmail.com (M.D.); EmilyFeuss@gmail.com (E.H.F.); slr2194@tc.columbia.edu (S.R.-H.); kelly.wen@my.liu.edu (K.W.); 2 Computing and Information Services, Vassar College, Poughkeepsie, NY 12604, USA; chgahn@vassar.edu

**Keywords:** coumarin, grapefruit effect, bergamottin, fraxidin, osthole, CYP3A4, crystal structure, DFT studies

## Abstract

Coumarins are plant-derived secondary metabolites. The crystal structure of three coumarins—bergamottin, osthole and fraxidin—are described and we analyze intermolecular interactions and their role in crystal formation. Bergamottin is a furanocoumarin found in citrus plants, which is a strong inhibitor of the principal human metabolizing enzyme, cytochrome P450 3A4 (CYP3A4). The crystal structure determinations of three coumarins give us the geometrical parameters and reveal the parallel-displaced π–π stacking and hydrogen bonding intermolecular interactions used for molecular assembly in the crystal structure. A quite strong (less than 3.4 Å) stacking interaction of bergamottin appears to be a determining feature that distinguishes it from other coumarins studied in this work. Our DFT computational studies on the three natural products of the same coumarin family docked into the active site of CYP3A4 (PDB 4D78) show different behavior for these coumarins at the active site. When the substrate is bergamottin, the importance of π-π stacking and hydrogen bonding, which can anchor the substrate in place, appears fundamental. In contrast, fraxidin and osthole show carbonyl coordination to iron. Our docking calculations show that the bergamottin tendency towards π–π stacking is important and likely influences its interactions with the heme group of CYP3A4.

## 1. Introduction

Coumarins are plant-derived secondary metabolites, with the parent compound being coumarin, chromen-2-one. As with other naturally derived compounds such as flavones, chalcones, etc., there are a large number and wide variety of structures due to potential substitution of hydrogen atoms with other functional groups. The biological activities of these compounds have been well studied and include competing with Vitamin K to produce the anticoagulant activity of coumarins such as warfarin [1].

**Scheme 1:**
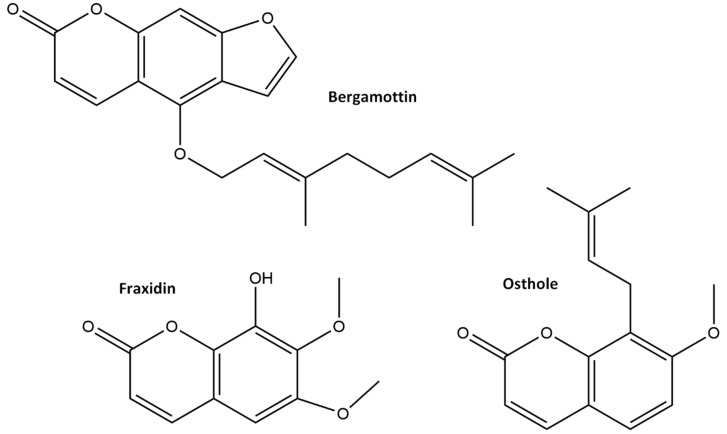
Coumarin compounds studied in this work.

Citrus plants produce coumarins and furanocoumarins and different citrus species show diversity in coumarin and furanocoumarin content. Interestingly, a 2015 study shows that pomelos, citrons and papedas synthesize high amounts of these compounds, whereas mandarins produce less [2]. The furanocoumarin, bergamottin (5-Geranoxypsoralen), Scheme 1, is a mechanism-based inhibitor of the Cytochrome P450 CYP3A4 enzyme, the major liver and intestinal P450 in humans. It is mainly responsible for the “grapefruit effect”, whereby the bioavailability of some pharmaceutical compounds is increased. CYP3A4 is the predominant member of the P450 family, a group of mixed-function oxidases that account for the metabolism of a majority of xenobiotic compounds as well as endogenous biochemicals such as cholesterol [3]. Bergamottin also inhibits other members of the P450 family such as CYP1A1, and to a lesser extent, CYP1A2.

A recent review highlights the structural features of cytochrome P450 enzymes which catalyze a large variety of oxidation reactions and which show an extraordinary flexibility in the dimensions of the P450 active site that depends on the chemical features of their substrates [4]. The coordinates of a large number of cytochrome P450 enzyme structures that have been studied are available from the Protein Data Bank (PDB) [5]. Observing those structures in the PDB with bergamottin or its derivatives at the active site, we note a crystal structure of CYP1A1 (PDB 6DWM) that shows the bergamottin geranoxy tail bent over the heme, while its psoralen ring system displays π–π interactions with Phe-224 of the F-helix [6]. A recent report of a crystal structure of CYP3A4 with 6′,7′-dihydroxybergamottin, DHB, a metabolite of bergamottin, bound at the active site, also exists (PDB 6OOB) [7]. The DHB psoralen ring lies above the heme and parallel to the I-helix, with the carbonyl oxygen H-bonded to the S119 hydroxyl group. Very recently, docking calculations on bergamottin, and a synthesized bergamottin derivative containing a nitroxide moiety and having antitumor effects, with CYP3A4 (PDB 2V0M), were performed [8].

We also investigated a C-prenylated coumarin-derivative, osthole (7-methoxy-8-(3-methyl-2-butenyl)-2*H*-1-benzopyran-2-one), Scheme 1, that shows neuroprotective, osteogenic, anticancer, hepatoprotective and potential antioxidant properties. Osthole is found in citrus peels [2] and in *Cnidium monnieri*, a flowering plant commonly used in traditional Chinese medicine. *C. monnieri* has been traditionally used to treat itching, inflammation, and other skin ailments [9].

The third coumarin studied is fraxidin (6,7-dimethoxy-8-hydroxycoumarin), Scheme 1. It can be found, among other natural sources, in the inner shell of the Japanese chestnut, *Castanea crenata*. It is of interest as a potential antidiabetic compound because of its insulin mimetic ability [10].

In this work, we describe the experimental results of single crystal X-ray structure determinations of three coumarin compounds as well as computational studies describing the docking of these coumarins into the active site of a CYP3A4 protein (PDB 4D78) after removal of ritonavir [11]. Single crystal studies are ideal for studying the packing between molecules which is dependent on the overall effect of all the intra- and intermolecular interactions in the crystal. The π–π stacking and hydrogen bonding are two important and significant forces in the interaction of molecules, regardless of whether they are a protein or a small molecule. The former interaction is not as simple to understand as hydrogen bonding and has been the subject of a recent review [12]. Janiak performed an early systematic and statistical investigation on stacking in metal–π–ligands by analyzing quantitative experimental crystal data found in the Cambridge Structural Database, CSD [13] and concluded that most interactions were parallel displaced and that atom–atom contact distances are an important measure to suggest the strength of π–π interactions with values around 3.3 Å (strong) > 3.6 Å (weaker) > 3.8 Å (weakest distance for which π–π interactions are acknowledged) [14]. Although weaker than H-bonding, the stabilization energy in these widely found interactions increases when there are heteroatoms in the rings. For example, the stacking energy in nucleic acid bases is 10–17 kcal/mol [15].

The purpose of the current study was to study how the crystal structure results of three different coumarin structures can assist us in probing CYP3A4 active site flexibility. We evaluate the relationship of the hydrogen bonding and interactions between conjugated systems in these three coumarin structures relative to the positions of these small molecules as they are docked into the CYP3A4 active site. In particular, we consider how the molecular structure of bergamottin can influence the active site molecular interactions and might induce the adverse physiological effect seen in many individuals upon eating grapefruit.

## 2. Materials and Methods

### 2.1. Materials

All materials were purchased from commercial sources. Bergamottin was from Glentham Life Sciences Ltd., (Wiltshire, UK) and Indofine Chemical Co. (Hillsborough, NJ, USA); fraxidin was from Indofine (Hillsborough, NJ, USA) and osthole was from Cayman Chemical Co. (Ann Arbor, MI, USA) and they were stored in the freezer (−20 °C).

### 2.2. Methods: Single Crystal X-ray Crystallography

Single crystal X-ray diffraction (XRD) was performed on bergamottin, osthole and fraxidin using a Bruker APEX2 DUO platform X-ray diffractometer. Single crystal studies give a fixed molecular structure, which is close to the lowest energy conformation. When a flexible region such as bergamottin’s geranoxy tail exists, the observed molecular conformation is influenced by the intermolecular interactions. All data measurements were taken at a temperature of 125 K and using Mo radiation. Crystal data for the three compounds are in Table 1. Structure solution and refinement was done using SHELX software [16]. Hydrogen atoms in bergamottin and osthole structures were found experimentally from difference Fourier electron density maps, while those in fraxidin were placed in calculated positions. Bergamottin and osthole were analyzed from crystals in the commercial samples, while fraxidin was recrystallized from ethanol.

Tables of atomic coordinates and isotropic displacement parameters for the three structures are reported in Tables S1–S3. Supplementary crystallographic data for this paper are deposited at the Cambridge Crystallographic Data Centre—Bergamottin CCDC no. 2008889; Osthole CCDC 2008888; Fraxidin CCDC 2008887. These data can be obtained free of charge via http://www.ccdc.cam.ac.uk/cgi-bin/catreq.cgi or from the Cambridge Crystallographic Data Centre, 12 Union Road, Cambridge, CB2 1EZ, UK; fax: (+44) 1223 336 033 or e mail: deposit@ccdc.cam.ac.uk.

### 2.3. Methods: Theory/Calculation, Docking and DFT Study

These calculations were performed using software programs from Biovia, San Diego, CA, USA. For docking, we used the CDOCKER [17] package in Discovery Studio 2017 version. The forcefield was CHARMm36, useful for metal calculations as the enzyme contains iron in the heme active site. CYP3A4 (PDB 4D78) [11] is a stored P450 set of atoms containing ritonavir as a guest; it was used as a receptor. We selected the S(ritonavir) atom as the center of the docking sphere of radius 8 Å and later eliminated ritonavir to perform docking of our several compounds. The docking output included 10 poses and to some of them, a standard dynamic cascade protocol was applied according to the default condition, consisting of minimizations, heating and production steps. All molecules treated were maintained in place after this procedure. Density functional theory code DMol3 was applied to calculate energy and geometry, implemented in Materials Studio 7.0 (PC platform) [18]. We employed the double numerical polarized (DNP) basis set that includes all the occupied atomic orbital plus a second set of valence atomic orbitals, and polarized *d*-valence orbitals [19], and correlation generalized gradient approximation (GGA) was applied including Becke exchange [20] plus Perdew correlation (BP) [21]. All electrons were treated explicitly and the real space cutoff of 5 Å was imposed for numerical integration of the Hamiltonian matrix elements. The self-consistent field convergence criterion was set to the root mean square change in the electronic density to be less than 10^−6^ electron/Å^3^. The convergence criteria applied during geometry optimization were 2.72 10^−4^ eV for energy and 0.054 eV/A for force. Dmol^3^ calculations included some protein residues that were truncated with the eliminated residues substituted by H atoms, which were kept fixed. However, those residues involved in specific potential bonding to the guest molecules were allowed to refine, and the truncated H replacing atoms were kept fixed.

## 3. Analysis

### 3.1. X-ray Crystal Structural Characterization of Bergamottin

**Figure 1:**
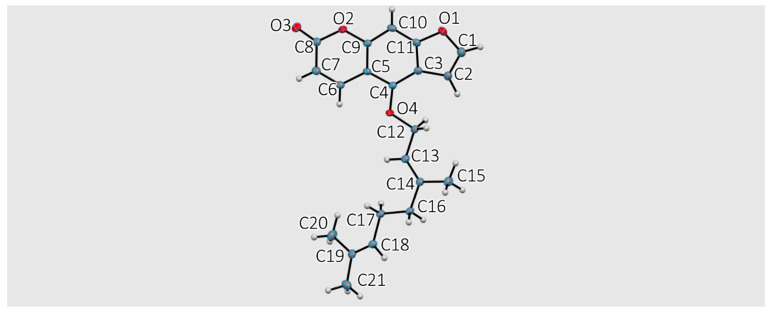
Labeling and bergamottin asymmetric unit in the crystal structure.

**Figure 2:**
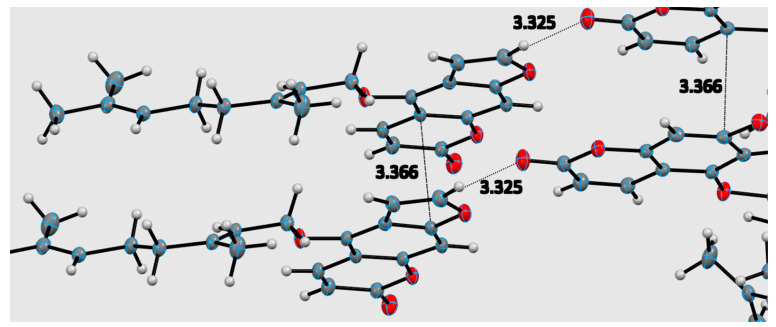
Stacking (offset) and hydrogen bonding distances in bergamottin crystal structure.

Obtaining crystals of bergamottin was attempted numerous times using a large variety of conditions with unfavorable results. Finally, a suitable crystal was found directly from a commercial sample and data were collected and analyzed at 125 K. Crystal data are reported in Table 1. There was some slight twinning but the amount of the second component was small and so data were refined using the major component only. The molecular structure shows no unusual features and the labeling and asymmetric unit are shown in Figure 1. The molecule can be considered to have two planes at about 38° to each other, as shown in Figure S1. One plane contains the three membered ring system while the other contains the geranoxy tail. The crystal structure shows two important intermolecular interactions seen in Figure 2. These consist of short offset stacking of the planar 3-ring system at 3.366 Å and a weak hydrogen bonded interaction between two bergamottin molecules involving the carbonyl oxygen (O3), C1-H1....O3 at 3.325 (4) Å and C-H-O bond angle of 166 (2)°, well within the van der Waals distance cutoff of 3.7 Å.

### 3.2. X-ray Crystal Structural Characterization of Osthole

**Figure 3:**
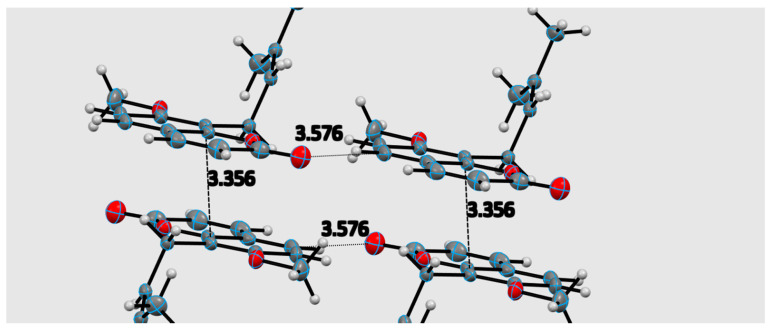
Hydrogen bonding and stacking interactions in osthole crystal structure.

The structure of osthole had been previously reported at room temperature [22]. The relationship between our cell parameters and the 1989 (CCDC code JAKFIK) structure is that ours is the reduced cell. Our crystal structure data for osthole are summarized in Table 1. The molecular structure of osthole shows no unusual features and is shown in Figure S2. The crystal structure shows intermolecular interactions with osthole having an offset stacking pattern, as shown in Figure 3. The molecule is bent at C10 with an angle of 74° between the planar aromatic coumarin ring system and the alkyl portion Figure S3. The offset stacking is similar to that seen in bergamottin, but at 3.366 Å and a weak C-H…O(carbonyl) hydrogen bond of 3.576 (3) Å and a C-H-O angle of 161 (1)°.

### 3.3. X-ray Crystal Structural Characterization of Fraxidin

**Figure 4:**
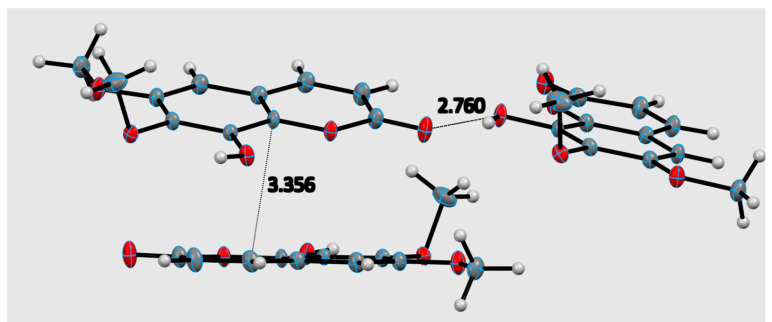
Stacking and hydrogen bonding interactions seen in the crystal structure of fraxidin.

Fraxidin crystal data are given in Table 1. The crystal structure of fraxidin shows two molecules in the asymmetric unit with little difference between them, shown in Figure S4. The molecules in the asymmetric unit do not show any deviations from expected bond distances and angle values. The fraxidin molecules are mostly planar except for the methoxy substituents (Figure 4) and in the crystal structure, they show important and strong intermolecular interactions. There is a strong hydrogen bond between two molecules of fraxidin O(5)-H(110)…O(9) at 2.760 (2) Å and angle 157.1 (4)°. There are also strong offset stacking interactions at 3.356 Å.

## 4. Theoretical Computational Studies

In order to study the interactions at the CYP3A4 active site, we perform docking studies using the crystal structure coordinates of our three coumarin structures placed into the active site of a CYP3A4 protein whose crystal structure coordinates are available from the Protein Data Bank. We chose the protein (PDB 4D78) because it was co-crystallized with a large drug molecule ritonavir at the active site. Ritonavir is a strong CYP3A4 inactivator [11]. Therefore, after the protein was “prepared” following the standard protocol, which includes H generation, the sulfur (ritonavir) atom was selected to establish the center of a sphere whose radius was 8 Å. Then, ritonavir was removed and bergamottin was docked using the program CDocker, which applies the molecular mechanic system CHARMm. Since the P450 protein contains the heme prosthetic group with an Fe metal iron, we employed CHARMm36 instead of using the default CHARMm setting. The resulting 10 configurations show two well distinct groups of interaction with the P450 heme. Poses 1–5 have the coumarin carbonyl coordinated to iron, in contrast with poses 6, 8–10 that show π–π co-planarity between the flat bergamottin aromatic rings and protein heme.

**Figure 5:**
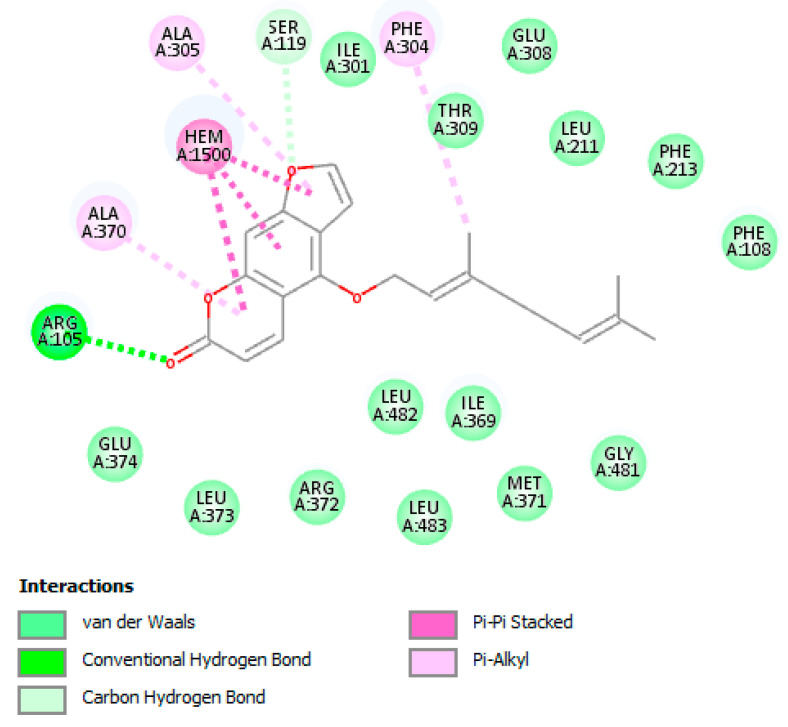
Berg (pose 6) 2D interactions, showing π–π display with the heme. There are also relevant interactions with Ser−119 and Arg−105. However, the large majority of residual action is of van der Waals type, and due to the hydrocarbon tail of bergamottin.

**Figure 6:**
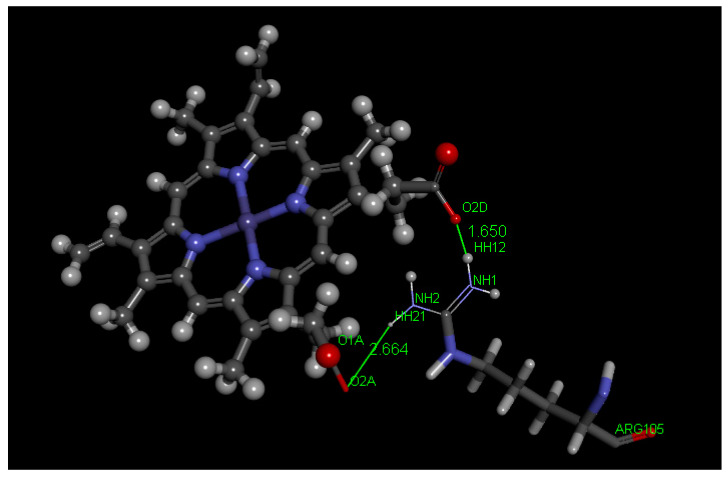
Heme stabilization in pose 6 is helped by hydrogen bonds to both terminal NH_2_ groups of Arg105.

**Figure 7:**
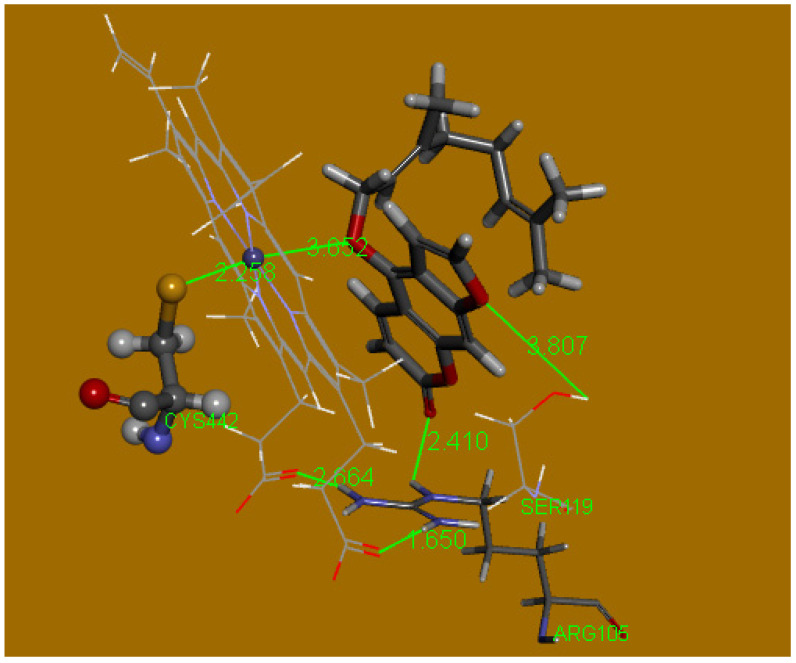
Pose 6 shows π–π co-planarity between the flat bergamottin ring system and the protein heme. Arg−105 establishes three additional important H-bond interactions: Arg−105 NH…O(coumarin carbonyl) and 2 H-bonds, with both heme carboxylates, through both terminal NH_2_ groups. S(Cys−442) completes the Fe coordination sphere from the protein. The geranoxy O(bergamottin) forms an angle of 151° with Fe, and is 3.652 Å apart, which may be thought to establish the 6th coordination site of the iron coordination sphere. A related DFT study of this system will be later described.

Figure 5 shows the protein amino acid residues interacting with bergamottin pose 6; a H(Ser−119) has a contact with furan ring O of bergamottin. This is described as a van der Waals type by the docking procedure which keeps the protein fixed, yet it is indeed a H-bond after free rotation by the corresponding serine moiety. Thus, after a serine torsion rotation from H-C-O-Hc 65.7° to 55°, [d[Hc-O(Berg6)]] becomes 2.59 Å. This may be an interesting specific interaction for bergamottin compared with other coumarins, see below. It should be noted that both terminal NH_2_ groups of Arg−105 are engaged with both P450 heme carboxylates through H-bonds (Figure 6 and Figure 7). However, bergamottin carbonyl in pose 6 also interacts with Arg−105 through the adjacent NH group. The role of Ser−119 and Arg−105 will also be explored using a quantum-mechanical approach.

**Figure 8:**
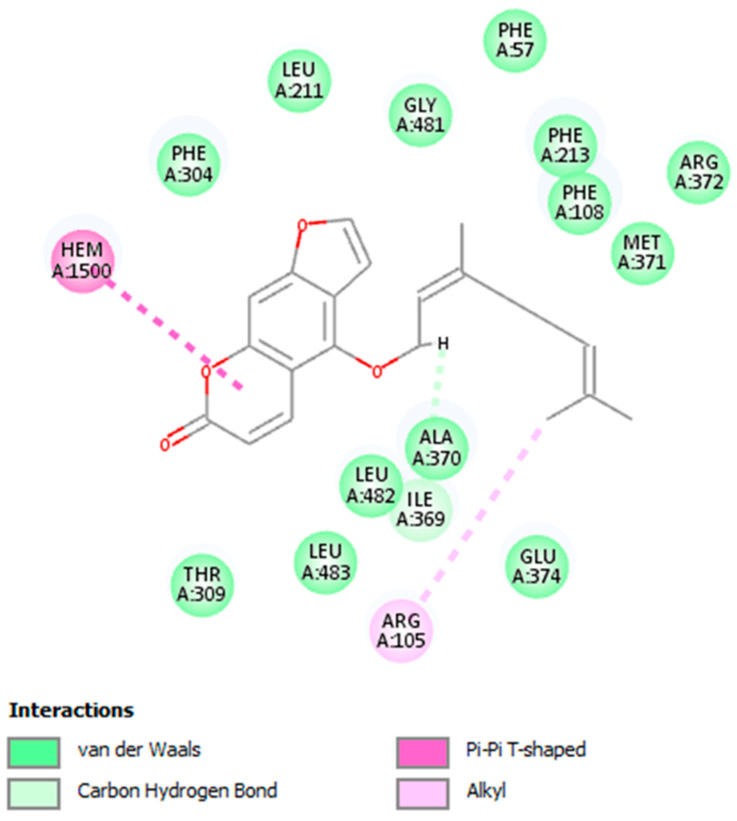
2D diagram for bergamottin–amino acid interactions from pose 5.

**Figure 9:**
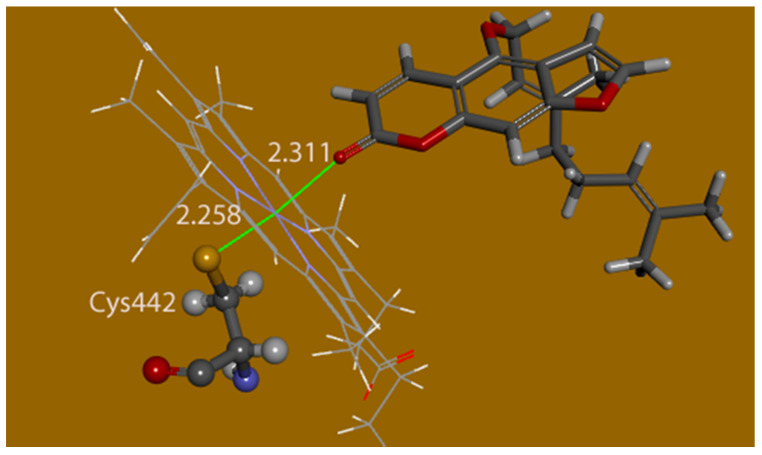
2D Docking of bergamottin pose 5 displaying an octahedral Fe configuration, having the same heme projection as Figure 7 shown earlier.

The protein amino acid residues interacting with bergamottin pose 5 are shown in Figure 8. Bergamottin pose 5 (Figure 9) shows the iron heme center displaying an octahedral configuration. Most of residual interactions are of the van der Waals type. In addition, bergamottin pose 4 shows the heme coordination sphere with the coumarin carbonyl pointing to Fe, and, in addition, a lone pair–π interaction between Ile−369 and the furan ring, 2.292 Å. This is interesting because it can clarify a specific role for bergamottin, compared with other coumarins missing the furan moiety.

**Figure 10:**
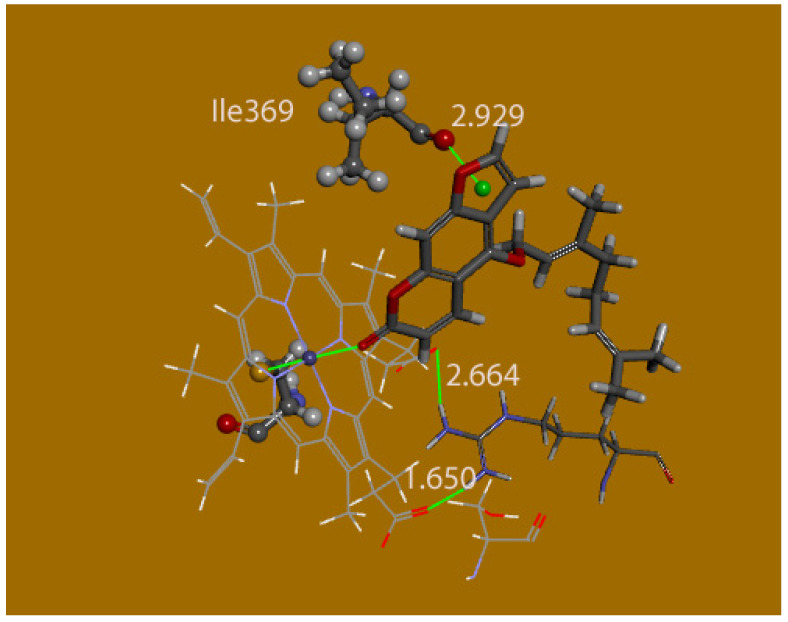
Bergamottin pose 4 showing Heme coordination sphere [d[Fe-O(carbonyl)]] = 2.292 Å, d[Fe-S = 2.258 Å, numbers omitted for clarity] and a lone pair–π interaction, 2.929 Å between Ile−369 and the furan ring. H-bonds regarding heme carboxylates and Arg105 are 1.650 and 2.664 Å.

The protein amino acid residues interacting with bergamottin pose 4 are shown in Figure S5. Bergamottin pose 4 (Figure 10) shows heme coordination sphere and a lone pair–π interaction between Ile−369 and the furan ring at 2.292 Å. This is noteworthy because it can clarify a specific role for bergamottin interaction.

Cdocker energy for poses 4, 5 and 6 of bergamottin are positive at 12.0, 12.1, and 12.4 kcal/mol, respectively. The CDocker interaction energies are all negative at −44.3, −41.8 and −38.4 kcal/mol respectively. A standard dynamic cascade was performed for the Berg6 conformer, without atomic restrictions, and the π–π arrangement was kept. For instance, the distance between the furan ring centroid and one C(heme) adjacent to one N(heme) is 3.815 Å. In contrast, the iron atom became displaced out of the N_4_ heme plane. This may be expected when a molecular mechanics program is used to describe a metal coordination sphere, and so a quantum-mechanical approach was employed, as shown below.

### 4.1. Quantum-Mechanical Calculations for Poses 6,5,4

**Figure 11:**
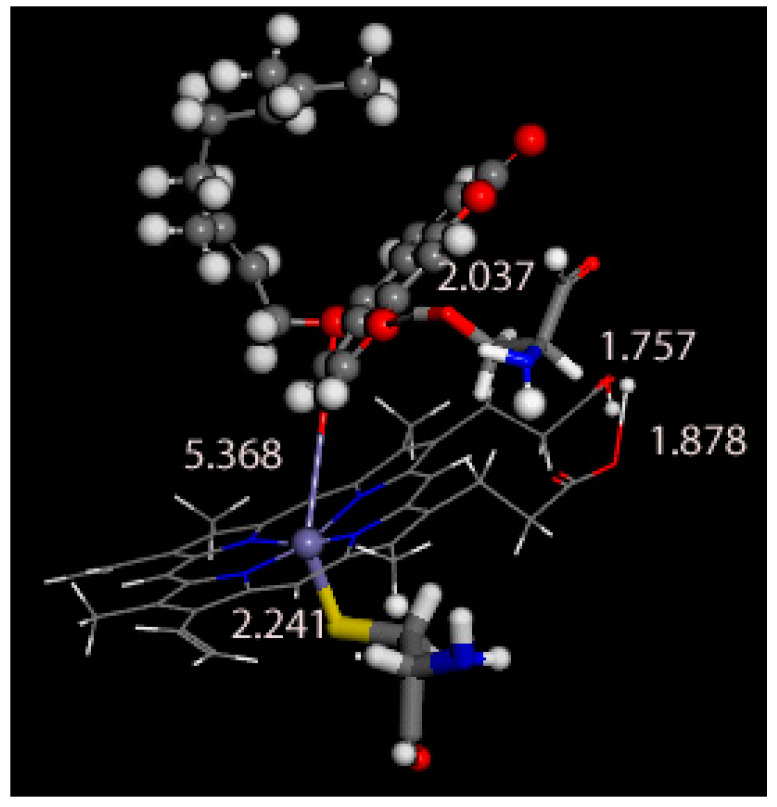
(**Berg6**). Result of DFT geometry minimization of docked Berg pose 6. All fixed amino acids are omitted for clarity. Arg−105 is only partially shown for clarity; only its 2 H atoms engaged in H-bonding with both heme carboxylates are depicted. These H-bonds O(carboxylate)-H(Arg−105) were 1.650 and 2.664 Å in the docked molecule, and now become 1.757 and 1.878 Å, respectively (shown). Ser−119 increases its attractive H-bond interaction with the Berg6 O(furan) atom, from 2.59 to 2.037 Å, and this contributes to anchoring Berg6 at the active site. In addition, after geometry minimization, Cys−442 does not modify substantially its bond to the iron center as the distance S-Fe, 2.258 Å in the docked molecule, becomes only slightly shorter at 2.241 Å. The heme profile seems to become slightly basket-like. Bergamottin O(carbonyl) does not confirm its interaction with NH(Arg−105) as the docked d[O(carbonyl)-HN] = 2.41 Å goes completely apart, 6.59 Å, not shown. On the whole, the minimization describes a slight shift of Berg6. Atom styles: bergamottin, ball and stick; heme, line; Ser−119, thin stick; Cys−442, thick stick.

**Berg6**: The input file was taken from the DS procedure, that is, the docked molecule of Berg6 was transferred into the Dmol3 program to perform geometry optimization. To conserve the protein environment, the following amino acid residues, Phe−302_Ile−303_Phe−304_Ala−305_Gly−306, were included in the DFT calculation, and their atomic coordinates were fixed. We also included some other residues, engaged in important H-bonds; these were less restrained. Thus, Ser−119, engaged in H-bonds with Berg6 and Arg−105 had the maximum freedom, that is, its connected residues Ile−118 and Ile−120 were replaced by H atoms, which were kept fixed. Arg−105 and Arg−106 were included and partially refined, that is, only the hydrocarbon chain of Arg−105 was not restricted, while their associated peptides Asn−104 and Pro−107 were substituted by H atoms, kept fixed. In addition, heme and Berg6 were not restricted at all. Figure S6 shows this initial situation. After minimization, the obtained structure confirms the π–π arrangement (Figure 11). However, some important changes are observed, for instance, the potential interaction between bergamottin ether oxygen with iron seen in Figure 7 was not confirmed, resulting in the lengthening of the corresponding Fe-O distance from 3.652 to 5.368 Å. Neither is the bergamottin carbonyl interaction with NH(Arg−105) seen after docking, as both atoms appeared completely apart at 4.782 Å. Cys−442 remains basically at the same distance from iron as in the docked molecule. The most important feature is shown by Ser−119 strongly bound to the O(furan) bergamottin, which may be important to explain bergamottin biological inhibitory action.

**Figure 12:**
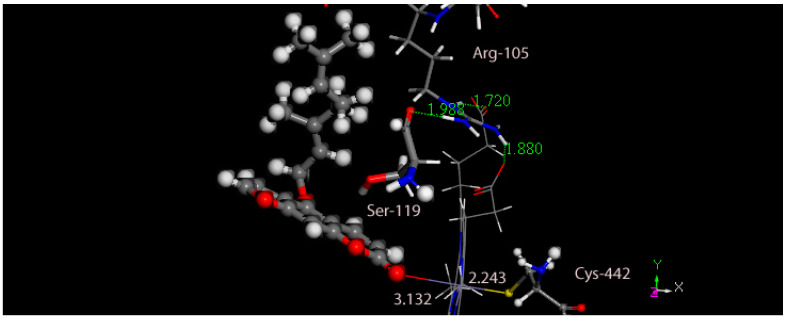
The main set of **Berg5** atoms after DFT geometry optimization with Dmol3 including the amino acid residue, Ser−119, located at the center (large stick style). Heme view is almost eclipsed and partially shown (line style); bergamottin (large ball and stick); Arg−105 partially shown (small stick); iron coordinated Cis−442 (small ball and stick). Iron confirms its bonding to S(Cys−442), 2.243 Å, however, bergamottin O(carbonyl) gets markedly removed from the heme coordination sphere, d(O-Fe) = 3.132 Å. Ser−119 remains linked via hydrogen bonding to Arg−105 NH_2_, 1.998 Å, as does Arg−105 to both heme carboxylates, 1.720 and 1.880 Å.

**Berg5**: The DFT geometry minimization of Berg pose 5 (Figure 12) also shows interesting features. The bergamottin O(carbonyl) moves markedly apart from the metal center at 3.132 Å; its initial separation after docking was much closer at 2.311 Å. After such shifting, Berg5 does not establish any other new interaction. Arg−105 and Ser−119 were treated the same way as was done with Berg6. This resulted in confirmation of important H-bond interactions. Thus, the Ser−119 H-bond to the NH_2_(Arg−105), which was 2.664 Å in the 4D78 protein, became strengthened to 1.998 Å. In addition, H-bonds seen between Arg−105 NH_2_ and NH groups to both heme carboxylates were confirmed at distances of 1.802 and 1.857 Å. Therefore, an important result of applying the DFT procedure to the docked molecular arrangement is the displacement of bergamottin from the heme coordination sphere, that is, the loss of the Fe-O(carbonyl) bond.

**Berg4**: The minimization of Berg4 shows the π–π interaction between the furan ring and the Ile−369 O(carbonyl) not confirmed, as the original distance of 2.292 Å becomes 5.59 Å. In addition, bergamottin O(carbonyl) bonding to the iron center is also lost, becoming d[O(carbonyl)-Fe] = 3.616 Å. We conclude that the active site interaction based on Berg4 docked configuration is not realistic.

The comparison of energies between Berg6 and Berg5 after Dmol application is of interest. Since both processes involved exactly the same molecules, including the same fixed atoms, this parameter may describe which configuration is preferred. The difference is 6.2 kcal/mol favoring Berg6, and so, the π–π configuration is energetically more stable. Moreover, as already described, the interaction between the furan oxygen with Ser−119 in Berg6 seems to provide another important element of consideration, as this is a specific structural feature of bergamottin, perhaps related to its characteristic biological activity, and so, we conclude that this π–π configuration for bergamottin suggests its inhibitory role of on CYP3A4, which may explain why bergamottin disturbs the metabolism of drugs, that is, it makes P450 be less available to perform its expected task [3].

#### 4.1.1. Comparison of Bergamottin with Other Coumarins

##### Fraxidin

**Figure 13:**
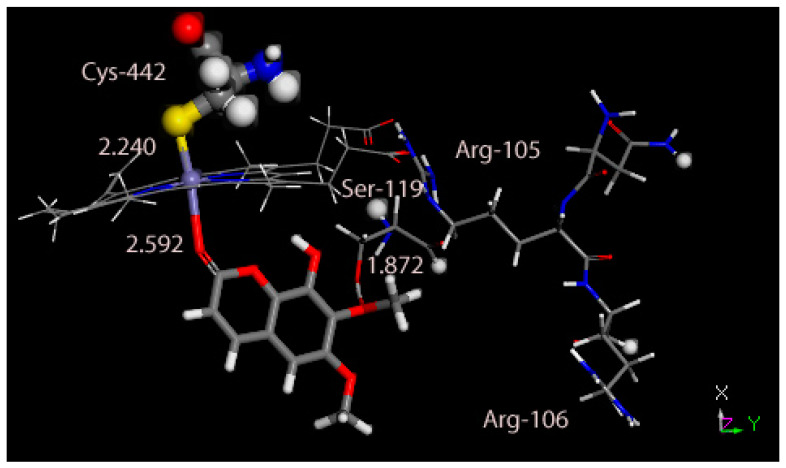
DFT optimized fraxidin pose 8 confirms Ser−119 H-bond to the fraxidin O(methoxy) group in position 7, 1.872 Å (for clarity, this bond interaction is not dashed) and octahedral iron coordination, O(carbonyl)-Fe = 2.592 Å and S(Cys-442)-Fe = 2.240 Å. Arg-105 keeps its H-bonds with both heme carboxylates, 1.807 Å (NH_2_) and 1.754 Å (NH) and Ser−119, 2.018 Å (NH_2_), with numbers omitted for clarity.

Fraxidin was docked using the same procedure described earlier for bergamottin. The 10 conformers always show an O(carbonyl) interaction with Fe. The most interesting conformer is pose 8, as seen by its 2D interactions below (Figure S7). This differs from the others by also having an H-bond with Ser−119. Therefore, its coordinates were input into the Dmol3 program to run a geometry minimization. This resulted in confirmation of the heme Fe-O(carbonyl) bonding (Figure 13).

#### 4.1.2. Osthole

Osthole was docked using the same procedure described earlier for bergamottin and fraxidin; of the 10 poses, pose 1, Ost1, and pose 3, Ost3, were selected for further study. The 2D interactions of pose 1 show O(carbonyl) bound to iron (Figure S8). The same diagram for Pose 3 has osthole O(carbonyl) engaged in 2 H-bonds and does not have a π-π interaction (Figure S9).

**Figure 14:**
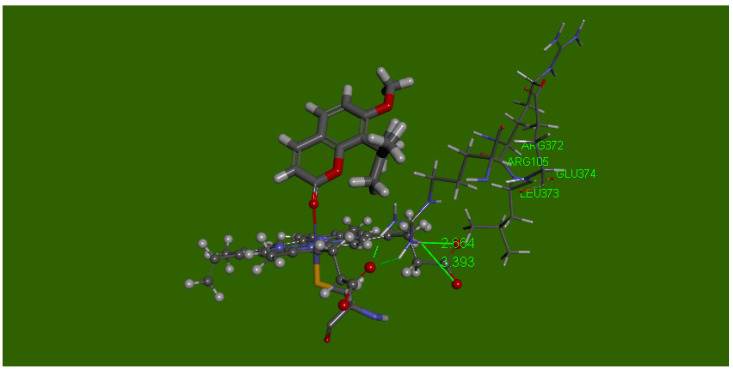
Pose 1 after docking of osthole with O(carbonyl) bound to iron, Ost1. This was selected to run DFT calculations.

**Figure 15:**
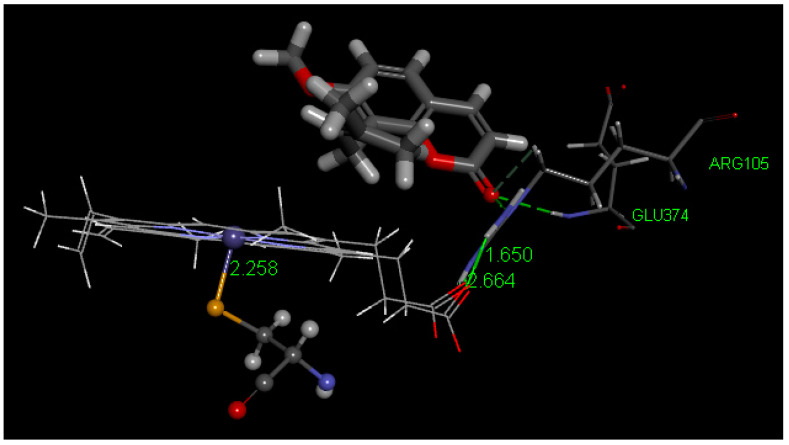
Pose 3 after docking of osthole was also selected to run DFT calculations.

**Figure 16:**
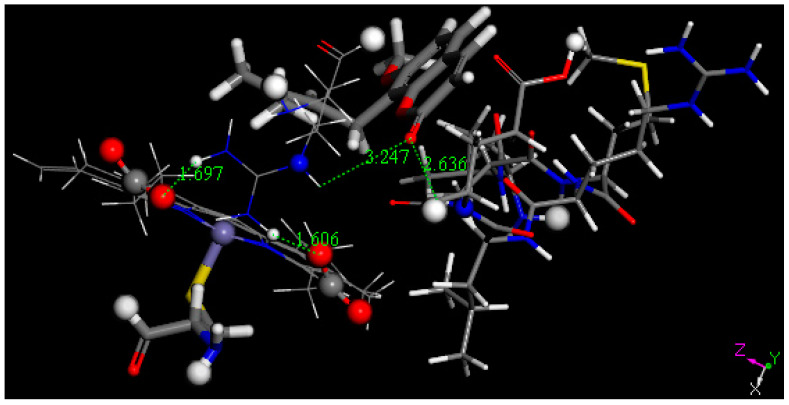
Docked pose 3 of osthole after DFT geometry optimization calculations.

The two configurations were geometry optimized with DFT methods and Ost3 was energetically more favored by 27.3 kcal/mol. The converged structures for pose 1 are shown in Figure 14 and Figure S10. The converged structures for the more stable pose 3, Ost3, are seen in Figure 15; Figure 16.

## 5. Results and Discussion

The crystal structures of three coumarin derivatives reveal their propensity for parallel-displaced π–π stacking, which is to be expected since coumarin contains an extended π-system. Short π–π distances imply strong charge transfer possibilities and makes them important contributors to the overall stability of the crystals. Additionally, in all three crystal structures, when proton donors are present, specific and directional hydrogen bonds are seen. Intermolecular interactions regulated by chemical functionality and molecular geometry thus, help manage molecular organization. These contacts help to identify potential molecular specificity or recognition and many chemical and biological pathways rely on their presence.

Taking advantage of the crystal structures of bergamottin, osthole and fraxidin, we inserted their atomic coordinates into a molecular mechanics docking procedure (Discovery Studio program) using CHARMm36 forcefield, specially designed to deal with metals such as iron, in the heme group at the enzyme active site. The selected receptor for this process was PDB 4D78, which contains coordinates for the CYP3A4 enzyme plus ritonavir, a potent enzyme inactivator [11]. We proceeded by docking bergamottin and found two distinct configurations. In one, there is an O(carbonyl)-Fe bond establishing an octahedral metal coordination, corresponding to bergamottin acting as a sixth ligand (pose 5). The other arrangement is described as π–π configuration involving the heme moiety (pose 6). Our work tried to differentiate between the two configurations.

Although energy is a discriminatory parameter, and the – system has a lower energy, suggesting it to be more stable as indicated by the CDocker interaction energy ∆E = 3.3 kcal/mol, we must proceed cautiously when comparing docking output results. Therefore, a standard dynamic cascade procedure was applied to both configurations, and some atoms at the active site showed substantial changes. For instance, the Fe atom became displaced out of the heme plane of pose 5 and the O(carbonyl)-Fe bond was lost, while in the π–π configuration (pose 6), there was only some shifting in the bergamottin position. In both configurations, a square-pyramidal coordination was shown by the iron center. Hence, a quantum-mechanical approach was used to discern the two docked configurations—one having the π–π arrangement, Berg6, and the other displaying bergamottin carbonyl coordinated to the Fe center, Berg5. Thus, we performed geometry optimizations for both configurations using the DFT program Dmol^3^. This program uses fewer parameters than those dealt with in a molecular mechanics program, and so, we had to restrain the protein environment. To save the active site conditions imposed on bergamottin, some amino acids were introduced in our calculation; the location of most of them implied van der Waals interactions and so, they were kept fixed to decrease calculation times. However, associated amino acids showing interactions such as H-bonds were allowed to vary in our calculation since these are very important. For instance, in both cases, the two heme carboxylate moieties are linked through H-bonds to Arg−105, and a substantial part of this amino acid was not fixed.

After operating with the two configurations, we reached the following conclusions:

1. In the pose 5 configuration, Berg5, the iron bonding to the coumarin carbonyl was lost after geometry optimization, and so, Fe-O(carbonyl) distance, which was initially 2.311 after docking, became 3.132 Å. It has to be noted that the whole protein system was kept in place, for instance, Arg−105 had H-bonds with heme carboxylates and also Ser−119 with Arg−104 amino groups were conserved. In addition, Berg5, after removal from the iron coordination sphere, did not establish alternative interactions.
2. On the other hand, Berg6, pose 6 conserved the π–π arrangement, even though a slight shift was generated by the geometry optimization. More importantly, the initial H-bond established between Ser−119 and O(furan) bergamottin was conserved. This may be important to explain bergamottin biological inhibitory action, i.e., this feature seems to be specific for bergamottin.

An additional arrangement, pose 4, was also analyzed quantum mechanically, but resulted not feasible. The difference in energy between poses 5 and 6 was marked, 6.2 kcal/mol favoring pose 6, and so, the π–π configuration is energetically more stable. It is interesting to compare our pose 6 configuration with a docking study of bergamottin [8], which used a different receptor (PDB 2V0M) derived from the crystal structure of CYP3Y4 together with inhibitor ketoconazole. After docking, a stacking configuration was also concluded. However, in contrast with our study, H-bonds between the hydrophilic amino acid (Thr309) and the furan oxygen were described. There was no Ser119 H-bonding with bergamottin. Our docking study used the default setting of 10 poses and since we were intrigued by these different outcomes, we performed an additional docking inquiry increasing to 40 poses; this confirmed no interaction involving Thr309. The location of Thr309 is essentially opposite to Ser119, as both amino acids are just across from the Fe atom. Thus, the distance between O(Thr309) (potentially involved in H bonds) and Fe is 6.18 Å, slightly shorter than that between Fe and O(Ser119) at 8.44 Å. We conclude that the different display for the stacking configuration obtained in both studies is the result of different spatial availability for bergamottin in the active site, after eliminating ketoconazole [8] or ritonavir [11] in our study.

Another interesting comparison can be made with the substrate 6′,7′-dihydroxybergamottin, DHB, which has been described recently in a CYP3A4 crystal structure, PDB 6OOB [7]. Here, a marked difference is a molecule of water coordinated to the Fe atom and the aromatic system of DHB positioned 3.6–5.2 Å above the heme (~15° tilt). Interestingly, Ser119 forms an H-bond with the coumarin carbonyl. It is obvious that the polar part of DHB plays an important role in situating the guest in the crystal. That bergamottin and DHB would interact differently at the active site is to be expected as this study presented some experimental binding differences between bergamottin and DHB at the active site based on the shape of the titration curves—sigmoidal for bergamottin and hyperbolic for DHB [7].

Only one other P450 crystal structure exists with bergamottin at the active site, CYP1A1 (PDB 6DWM) [6]. In that situation, bergamottin’s aromatic ring system is involved in π–π interactions with Phe−224 of the F-helix (and not with the heme group), while its geranoxy tail is bent over the heme with the terminal 6′,7′ double bond lying offset from the heme iron at distances of 4.8 and 5.1 Å. From the placement and interactions of bergamottin at the active site, the authors suggest that CYP1A1 is likely to metabolize bergamottin into the 6′,7′-dihydroxy metabolite, DHB.

Therefore, the docking and DFT treatment of bergamottin, according to our study, seems to be consistent with the biologically important property of P450 inhibition. Bergamottin is found in the active site with the planar psoralen ring system stacked above the heme and with the furan oxygen atom about 4 Å distance from the heme iron, in contrast to the bergamottin location seen in two crystal structures described earlier. That is, the π–π display has lower energy than the Fe-carbonyl coordinated configuration. In addition, our π–π arrangement is closely related to the one established by the strong inactivator ritonavir [11], as in both cases, no Fe-O(carbonyl) bond is seen. Furthermore, the Fe-O pose within bonding distance, pose 5, was not confirmed by DFT, suggesting it is nonexistent. To our knowledge, there are no other structural studies of P450 protein crystal structures with other coumarins, except those described earlier. None show any Fe-O(carbonyl) bonding.

When reviewing the literature for fraxidin, we found no studies describing its interaction with the P450 enzymes. Our computational studies of 10 docked configurations always show an O(carbonyl) interaction with Fe, as was seen for Berg6. The most interesting conformer is Fraxi8, as it differs from the others by also incorporating an H-bond between Ser−119 and [O(7-methoxy)]. The quantum-mechanical approach confirmed this H-bond as well as the Fe-O(carbonyl) bond. Osthole docking studies confirm Fe-O(carbonyl) coordination plus hydrogen bonding as the lowest energy configuration.

**Figure 17:**
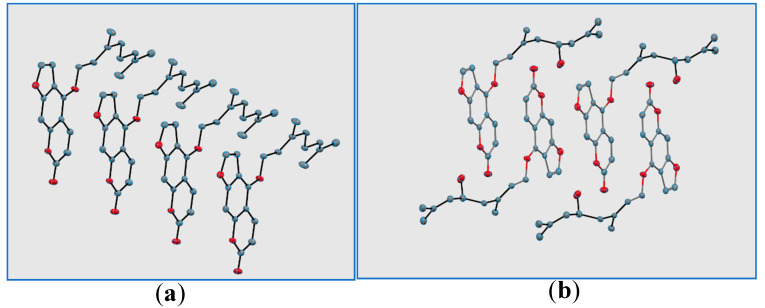
Crystal structure packing, hydrogen atoms omitted. (**a**) Bergamottin (this work); (**b**) Notopterol (CSD-SOLXUN) [22].

These results led us to infer that the distinguishing feature of the bergamottin molecular structure that permits such a strong inhibition reaction with CYP3A4 was the very strong stacking interaction. We searched the Cambridge Crystallographic Database (CSD) [13] for other furanocoumarin natural products and saw that presence of the O(ether) functional group played a major role in their stacking distance. In fact, furanocoumarin (CSD-FURCOU), with no substituent at all, had a stacking distance of 3.5 Å. Furanocoumarins with 8-O(ether) tail substituents (across from bergamottin’s) showed weaker stacking 3.7–3.8 Å (CSD-TOPXIH, CSD-YALZES). On the other hand, furanocoumarins with an 5-O(ether) tail in the same location as bergamottin, oxypeucedanin (CSD-PEXXAS), isoimperatorin (CSD-NUVHER), imperatorin (CSD-YAGHOE), and pranferol (CSD-KAHGEI), all had strong stacking between the furanocoumarin ring structure, around 3.4 Å. In particular, a natural furanocoumarin whose molecular structure is strikingly similar to bergamottin, notopterol (CSD-SOLXUN), was catalogued [23]. This compound is a widely used component of Chinese herbal medicine and its molecular structure is the same as bergamottin, except for a hydroxyl substitution at 5′. The comparison of the crystal packing for bergamottin and notopterol is shown in Figure 17.

It is clear that the extra polar 5′-OH group in notopterol causes a different type of packing (head–tail) than that seen in bergamottin (head–head). However, in both compounds, the furanocoumarin three-ring system has a very short stacking distance at 3.34 Å. With this similitude, we would expect notopterol to also be a strong inactivator of cytochrome P450 enzymes, and, indeed, the literature studies show it to be a strong inhibitor of CYP3A4 [24]. Additionally, a very recent article [25] demonstrated that notopterol inhibits around 94% of CYP2D6 activity, followed by CYP2B6 (77%) and CYP3A4 (50%).

## 6. Conclusions

**Figure 18:**
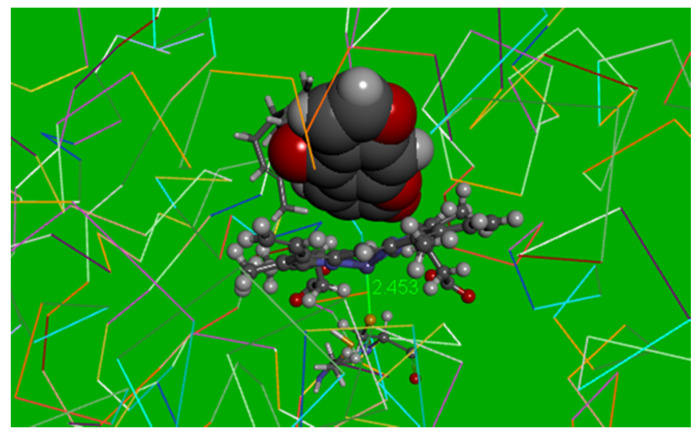
Output of the standard dynamic cascade of bergamottin pose 6 interaction with CYP3A4, showing iron coordination number 5 (the iron is out of the heme plane). The CPK planar moiety is bergamottin. The protein is in the C-alpha wireframe.

We wanted to probe the important structural features involved in bergamottin’s binding to CYP3A4, since it is a strong mechanism-based inhibitor. Its physiological role in grapefruit–drug interactions with many pharmaceutical compounds can modulate the bioavailability of these medications [3]. The small molecule crystal structure determinations of three coumarins of very different sizes—bergamottin, fraxidin and osthole—give us the geometrical parameters and reveal the parallel-displaced π–π stacking and hydrogen bonding intermolecular interactions used for molecular assembly in the crystal structure. Our computational studies on the three natural products of the same coumarin family docked into the active site of CYP3A4 (PDB 4D78) show different behavior at the active site. When the substrate is bergamottin, the importance of π–π stacking and hydrogen bonding, which can anchor the substrate in place appear fundamental. Figure 18 shows the standard dynamic cascade output of bergamottin docked pose 6 binding to CYP3A4. This process induces only a slight shift of the bergamottin from the initial docked position (Figure 7).

In contrast, fraxidin shows only carbonyl coordination to the iron. Osthole docking results show carbonyl coordination to the heme iron as well. We note that in the single crystal structure, a quite strong (less than 3.4 A) stacking interaction of bergamottin appears to be a determining feature that distinguishes it from other coumarins studied in this work and likely influences its interactions with CYP3A4. Our docking calculations show that the psoralen π–π stacking configuration with the heme group is important. Experimental studies show that there is no evidence for bergamottin covalent binding to heme iron [3]. Using computational approaches to predict substrate binding at the active site gives useful information about the substrate orientation and potential amino acid interactions.

## Figures and Tables

**Table 1:** X-ray Data for Bergamottin, Osthole and Fraxidin. All data were collected at Temperature = 125(2) K and (Mo) λ = 0.71073 Å.

Name	Bergamottin	Osthole	Fraxidin
Empirical Formula	C_21_ H_22_ O_4_	C_15_ H_16_ O_3_	2(C_11_ H_10_ O_5_)
Formula Weight	338.38	244.28	2(222.19)
Crystal System	Monoclinic	Triclinic	Monoclinic
Space Group	P2_1_/n	P-1	P2_1_/n
Unit Cell Dimensions	a = 4.3910(6) Å α = 90°	7.4543(11) Å 65.145(3)°	16.8409(16) Å 90°
	b = 25.704(4) Å β = 92.212(2)°	9.4107(14) Å 74.836(3)°	7.4773(7) Å 113.842(1)°
	c = 15.204(3) Å γ = 90°	10.7594(16) Å 70.553(3)°	16.8475(16) Å 90°
Volume	1714.7(4) Å^3^	639.23(17) Å^3^	1940.5(3) Å^3^
Z	4	2	4
Absorption Coefficient	0.090 mm^−1^	0.088 mm^−1^	0.122 mm^−1^
Crystal size	0.200 × 0.080 × 0.040 mm^3^	0.280 × 0.190 × 0.050 mm^3^	0.32 × 0.16 × 0.07 mm^3^
Θ range data collection	1.584 to 30.033°	2.107 to 32.054°	2.22 to 30.53°
Reflections Collected	42,708	16,847	31,103
Independent Reflections	5012 [R(int) = 0.0573]	4224 [R(int) = 0.0273]	5913 [R(int) = 0.0440]
Completeness to Θ = 25.242°	99.9%	100.0%	99.8%
Absorption correction	Empirical	Empirical	None
Data/restraints/parameters	5012/0/314	4224/0/228	5913/0/302
Goodness of fit on F^2^	1.184	1.034	1.068
Final R indices [I > 2 σ(I)]	R1 = 0.0848, wR2 = 0.2431	R1 = 0.0425, wR2 = 0.1169	R1 = 0.0498, wR2 = 0.1264
R indices (all data)	R1 = 0.1058, wR2 = 0.2547	R1 = 0.0560, wR2 = 0.1276	R1 = 0.0577, wR2 = 0.1344
Largest diff peak and hole	0.672 and −0.329 e.Å^−3^	0.417 and −0.248 e.Å^−3^	0.741 and −0.325 e.Å^−3^

## Supplementary Materials

The following are available online. Figure S1–S4: X-ray diffraction molecular structure results of Bergamottin, Fraxidin and Osthole, Figure S5–S10: Results of computational calculations, Tables S1–S3: Bergamottin, Osthole, Fraxidin atomic coordinates and equivalent isotropic displacement parameters. Supplementary crystallographic data for this paper are deposited at the Cambridge Crystallographic Data Centre. Bergamottin CCDC no. 2008889; osthole CCDC 2008888; fraxidin CCDC 2008887. These data can be obtained free of charge via http://www.ccdc.cam.ac.uk/cgi-bin/catreq.cgi or from the Cambridge, Crystallographic Data Centre, 12 Union Road, Cambridge, CB2 1EZ, UK; fax: (+44) 1223 336 033 or e mail: deposit@ccdc.cam.ac.uk.
