# Supplementary material for: The Grapefruit Effect: Interaction between Cytochrome P450 and Coumarin Food Components, Bergamottin, Fraxidin and Osthole. X-ray Crystal Structure and DFT Studies
Source: Molecules. 2020 Jul 10;25(14):3158. doi: 10.3390/molecules25143158 (PMC7397038; doi:10.3390/molecules25143158)
Supplement: Supplementary file 1 [file molecules-25-03158-s001.pdf]

The Grapefruit Effect: Interaction between Cytochrome P450 and Coumarin Food Components, Bergamottin, Fraxidin and Osthole. X-ray Crystal Structure and DFT Studies.

Miriam Rossi [1,*], Sandjida Aktar [1], Marissa Davis [1], Emily Hefter Feuss [1], Samara Roman-Holba [1], Kelly Wen [1], Christopher Gahn [2] and Francesco Caruso [1,*]

[1] Vassar College, Chemistry Department, Poughkeepsie, NY 12604, USA; sandjidaaktar123@gmail.com (S.A.); rissacldavis@gmail.com (M.D.); EmilyFeuss@gmail.com (E.H.F.); slr2194@tc.columbia.edu (S. R-H.); kelly.wen@my.liu.edu (K.W.).

[2] Vassar College, Computing and Information Services, Poughkeepsie, NY 12604, USA; chgahn@vassar.edu.

* Correspondence: rossi@vassar.edu (M.R.); caruso@vassar.edu (F.C.).

Supplementary Material

1. *Crystallographic Data*

Supplementary crystallographic data for this paper are deposited at the Cambridge Crystallographic Data Centre. Bergamottin CCDC no. 2008889; osthole CCDC 2008888; fraxidin CCDC 2008887. These data can be obtained free of charge via http://www.ccdc.cam.ac.uk/cgi-bin/catreq.cgi or from the Cambridge Crystallographic Data Centre, 12 Union Road, Cambridge, CB2 1EZ, UK; fax: (+44) 1223 336 033 or e mail: deposit@ccdc.cam.ac.uk.

2. *Supplementary Tables S1-S3*

Table S1. **BERGAMOTTIN** Atomic coordinates (x 10^4) and equivalent isotropic displacement parameters (Å^{2}x 10^3). U(eq) is defined as one third of the trace of the orthogonalized U^{ij} tensor.

	x	y	z	U(eq)
O(4)	2036(5)	6140(1)	2330(1)	21(1)
O(2)	2144(5)	7983(1)	2527(1)	20(1)
O(1)	-4252(5)	7142(1)	360(1)	22(1)
O(3)	5061(5)	8390(1)	3520(2)	27(1)
C(4)	848(6)	6592(1)	2002(2)	16(1)
C(3)	-1321(6)	6644(1)	1306(2)	17(1)
C(5)	2021(6)	7044(1)	2420(2)	16(1)
C(9)	1024(6)	7537(1)	2125(2)	16(1)
C(11)	-2165(6)	7153(1)	1052(2)	18(1)
C(6)	4209(7)	7026(1)	3149(2)	18(1)
C(14)	3799(7)	4795(1)	1962(2)	18(1)
C(17)	6680(7)	4486(1)	3384(2)	19(1)
C(10)	-1077(6)	7609(1)	1435(2)	19(1)
C(19)	9294(7)	3961(1)	4606(2)	19(1)
C(13)	2958(7)	5235(1)	2350(2)	18(1)
C(8)	4242(7)	7974(1)	3227(2)	19(1)
C(16)	5605(7)	4374(1)	2438(2)	19(1)
C(2)	-3090(7)	6308(1)	711(2)	20(1)
C(18)	8571(6)	4044(1)	3760(2)	18(1)
C(12)	1074(7)	5657(1)	1924(2)	19(1)
C(7)	5231(7)	7466(1)	3541(2)	21(1)
C(1)	-4752(7)	6620(1)	172(2)	22(1)
C(15)	3003(9)	4660(1)	1017(2)	28(1)
C(21)	11168(8)	3498(1)	4902(2)	27(1)
C(20)	8364(10)	4316(1)	5335(2)	31(1)
H(12B)	-1200(70)	5623(12)	1990(20)	7(7)
H(2)	-3000(80)	5922(14)	670(20)	16(8)
H(1)	-6190(90)	6556(15)	-290(30)	24(9)
H(6)	4990(100)	6700(17)	3340(30)	37(11)
H(7)	6580(90)	7460(14)	4010(30)	22(9)

H(10)	-1710(100)	7938(16)	1240(30)	34(11)
H(12A)	1450(80)	5679(14)	1300(30)	22(9)
H(13)	3490(80)	5309(13)	2970(20)	17(8)
H(15A)	5060(100)	4616(17)	750(30)	37(11)
H(15C)	1830(110)	4350(20)	970(30)	49(13)
H(15B)	1850(100)	4919(18)	690(30)	40(12)
H(16B)	4420(80)	4060(14)	2430(20)	18(8)
H(16A)	7520(90)	4309(15)	2130(30)	29(10)
H(17A)	4860(90)	4544(15)	3710(30)	30(10)
H(17B)	7890(90)	4787(15)	3410(30)	26(10)
H(18)	9370(80)	3800(14)	3320(20)	22(9)
H(20A)	7530(110)	4123(19)	5820(30)	50(14)
H(20B)	10220(120)	4480(20)	5560(30)	56(15)
H(21B)	13190(110)	3602(18)	5170(30)	46(13)
H(21A)	11810(100)	3284(18)	4380(30)	42(12)
H(21C)	10130(100)	3277(17)	5310(30)	35(11)
H(20C)	6830(130)	4580(20)	5170(40)	63(16)

Table S2. **OSTHOLE** Atomic coordinates (x 10^4) and equivalent isotropic displacement parameters ($Å^2$x 10^3)**.** U(eq) is defined as one third of the trace of the orthogonalized U^{ij} tensor.

	x	y	z	U(eq)
O(1)	4982(1)	2654(1)	5294(1)	23(1)
O(2)	5777(1)	3408(1)	6725(1)	37(1)
O(3)	3764(1)	933(1)	2156(1)	29(1)
C(1)	4351(1)	1790(1)	3725(1)	20(1)
C(2)	3009(1)	1513(1)	3203(1)	23(1)
C(3)	1040(1)	1826(1)	3733(1)	29(1)
C(4)	395(1)	2426(1)	4792(1)	30(1)
C(5)	1683(1)	2720(1)	5343(1)	25(1)
C(6)	1138(2)	3304(1)	6465(1)	32(1)
C(7)	2464(2)	3523(2)	6961(1)	35(1)
C(8)	4489(2)	3213(1)	6368(1)	28(1)
C(9)	3644(1)	2395(1)	4787(1)	21(1)
C(10)	6455(1)	1497(1)	3123(1)	20(1)
C(11)	6858(1)	3022(1)	1969(1)	20(1)
C(12)	7466(1)	3237(1)	628(1)	22(1)
C(13)	7896(2)	1962(2)	24(1)	34(1)
C(14)	7732(2)	4860(1)	-415(1)	33(1)
C(15)	2466(2)	663(2)	1556(2)	38(1)
H(3)	130(30)	1630(20)	3359(19)	46(5)
H(4)	-970(30)	2650(20)	5153(17)	42(4)
H(6)	-220(30)	3550(20)	6858(19)	45(4)
H(7)	2200(30)	3920(30)	7710(20)	60(5)
H(11)	6550(20)	3958(17)	2256(15)	26(3)
H(100)	7230(20)	1154(18)	3874(15)	31(3)
H(101)	6840(20)	567(19)	2809(16)	36(4)
H(130)	9260(30)	1750(20)	-418(19)	50(5)
H(131)	7650(30)	900(20)	740(20)	52(5)
H(132)	7160(30)	2330(20)	-710(20)	56(5)
H(140)	9100(30)	4800(20)	-835(18)	47(4)
H(141)	6960(30)	5220(20)	-1200(20)	51(5)

H(142)	7330(20)	5660(20)	-12(17)	41(4)
H(150)	1490(20)	1690(20)	1160(17)	38(4)
H(151)	1870(30)	-220(20)	2222(19)	48(5)
H(152)	3290(30)	300(20)	783(19)	49(5)

Table S3. **FRAXIDIN** Atomic coordinates (x 10^4) and equivalent isotropic displacement parameters ($Å^2$x 10^3). U(eq) is defined as one third of the trace of the orthogonalized U^{ij} tensor.

	x	y	z	U(eq)
O(1)	4629(1)	2634(2)	6614(1)	14(1)
O(2)	320(1)	2727(2)	6109(1)	22(1)
O(3)	1705(1)	2669(2)	6325(1)	15(1)
O(4)	5233(1)	3235(2)	8346(1)	20(1)
O(5)	2871(1)	2330(2)	5623(1)	16(1)
O(6)	1714(1)	7751(2)	4724(1)	15(1)
O(7)	1417(1)	7635(2)	1806(1)	14(1)
O(8)	3461(1)	8304(2)	5312(1)	18(1)
O(9)	1207(1)	7611(2)	428(1)	20(1)
O(10)	713(1)	7351(2)	2971(1)	17(1)
C(1)	1349(1)	3200(2)	7553(1)	20(1)
C(2)	2196(1)	3360(2)	8087(1)	18(1)
C(3)	2857(1)	3169(2)	7747(1)	15(1)
C(4)	3751(1)	3311(2)	8273(1)	17(1)
C(5)	4352(1)	3125(2)	7905(1)	15(1)
C(6)	4055(1)	2804(2)	7005(1)	13(1)
C(7)	4882(1)	4373(2)	6413(1)	26(1)
C(8)	1075(1)	2861(2)	6640(1)	17(1)
C(9)	2577(1)	2823(2)	6865(1)	13(1)
C(10)	5542(1)	3490(2)	9266(1)	21(1)
C(11)	3174(1)	2640(2)	6483(1)	12(1)
C(12)	1678(1)	5962(2)	5012(1)	26(1)
C(13)	2104(1)	7859(2)	4147(1)	14(1)
C(14)	1577(1)	7678(2)	3270(1)	13(1)
C(15)	1959(1)	7823(2)	2673(1)	14(1)
C(16)	1734(1)	7737(2)	1178(1)	16(1)
C(17)	2664(1)	7993(2)	1454(1)	19(1)
C(18)	4377(1)	8659(2)	5605(1)	20(1)
C(19)	3008(1)	8173(2)	4439(1)	14(1)
C(20)	3374(1)	8314(2)	3838(1)	16(1)
C(21)	2850(1)	8127(2)	2951(1)	14(1)

C(22)	3196(1)	8195(2)	2296(1)	18(1)
H(1)	923	3314	7786	24
H(9)	2360	3599	8686	22
H(8)	3944	3534	8877	21
H(2)	5178	5049	6951	39
H(6)	4363	5029	6033	39
H(7)	5274	4219	6118	39
H(4)	5363	2476	9523	31
H(5)	5298	4597	9384	31
H(3)	6177	3571	9518	31
H(14)	1383	5189	4508	39
H(10)	1358	5952	5385	39
H(15)	2270	5521	5340	39
H(11)	2902	8018	1029	23
H(17)	4653	7684	5421	29
H(12)	4639	8747	6240	29
H(18)	4462	9787	5354	29
H(16)	3979	8535	4027	19
H(13)	3800	8382	2459	21
H(140)	567(19)	7400(30)	3322(18)	33(7)
H(110)	3240(20)	2370(50)	5460(20)	60(10)

3. Supplementary Figures S1-S10

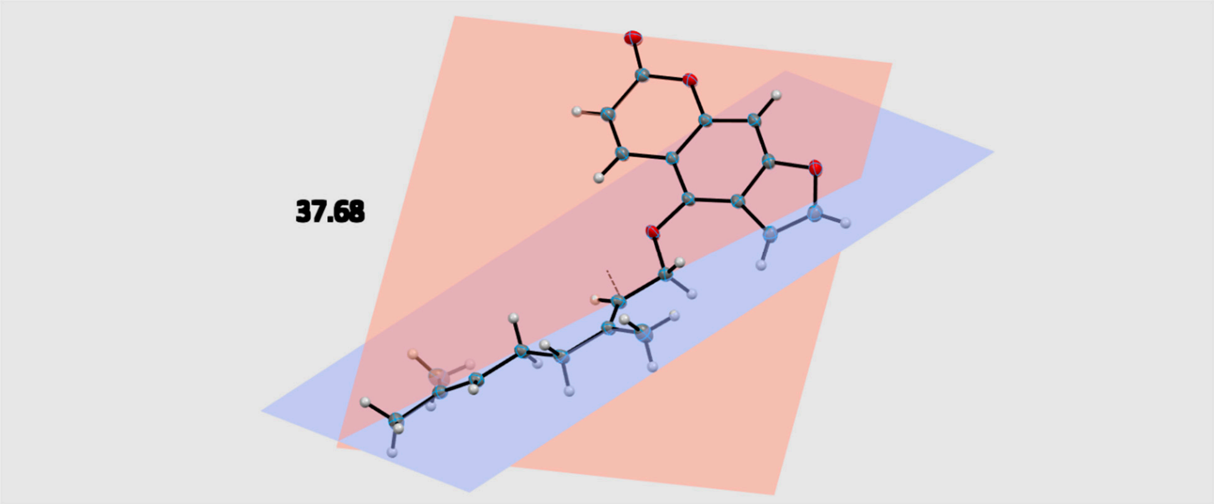

Figure S1. Two planes in bergamottin molecular structure can be seen with the angle between planes.

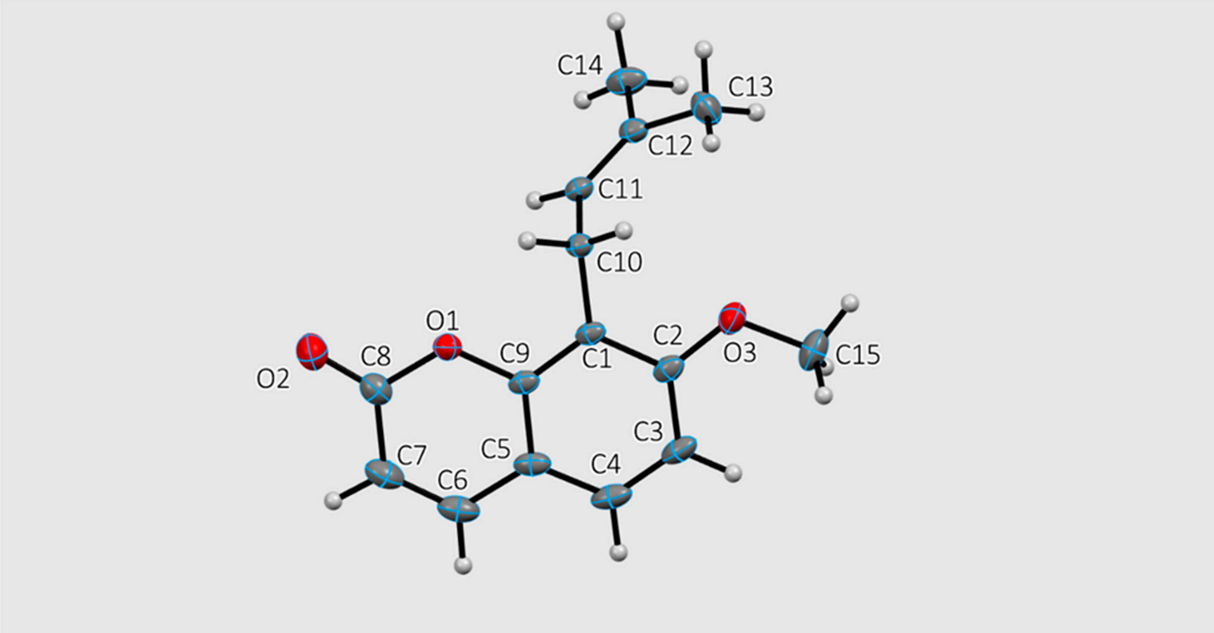

Figure S2. Single molecule of Osthole in asymmetric unit.

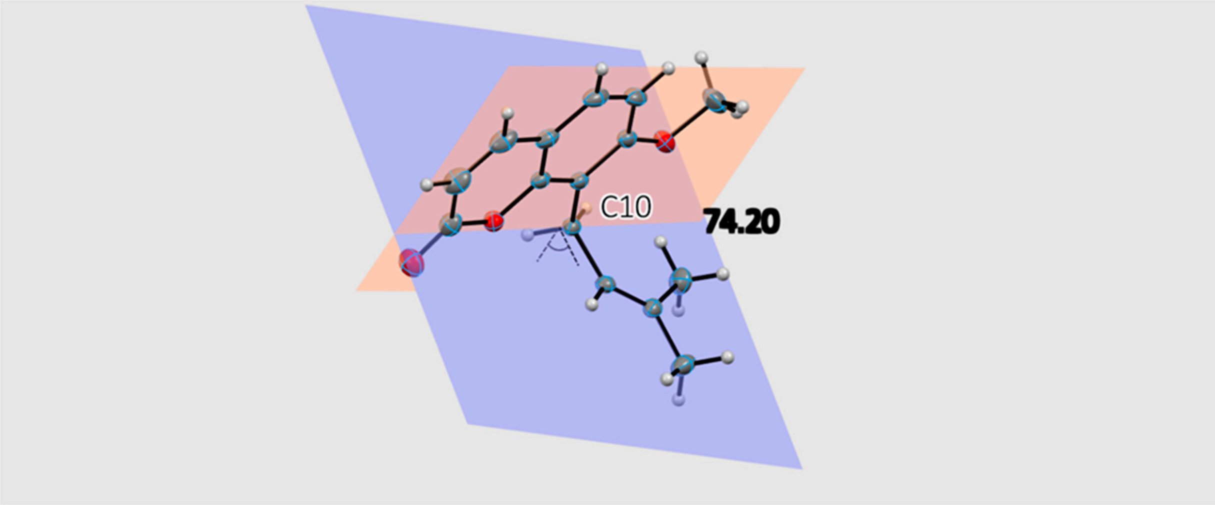

Figure S3. Angle between two planes of osthole molecular structure.

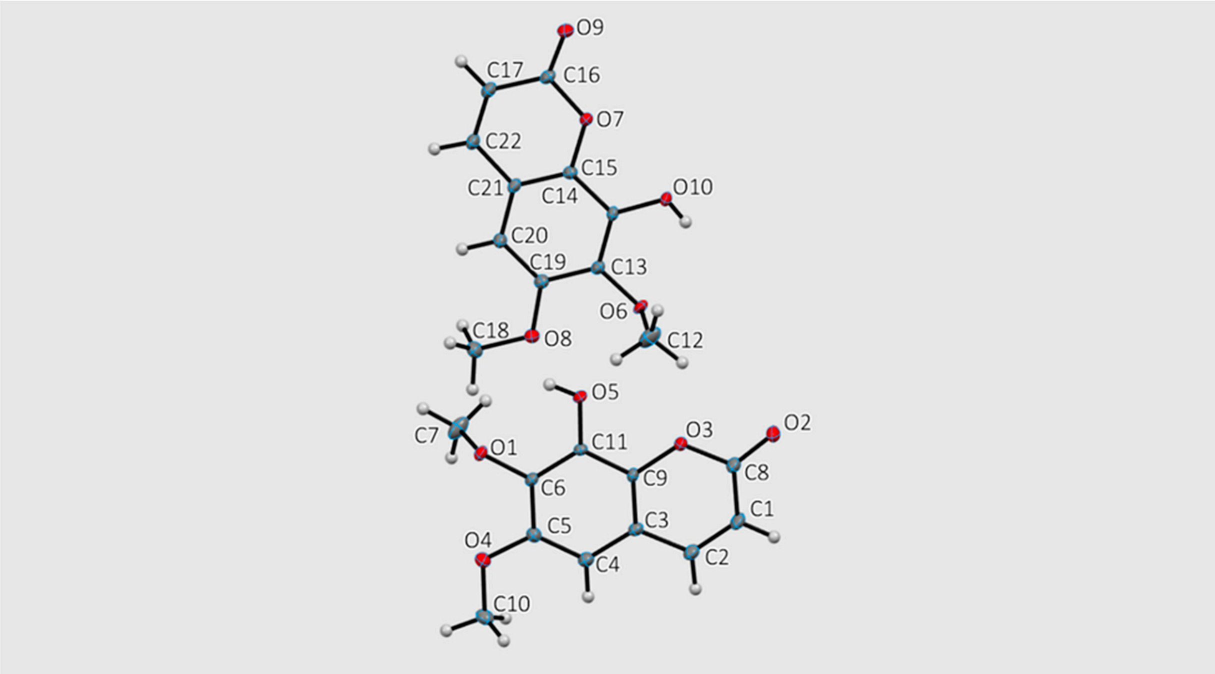

Figure S4. The two molecules in the asymmetric unit of fraxidin.

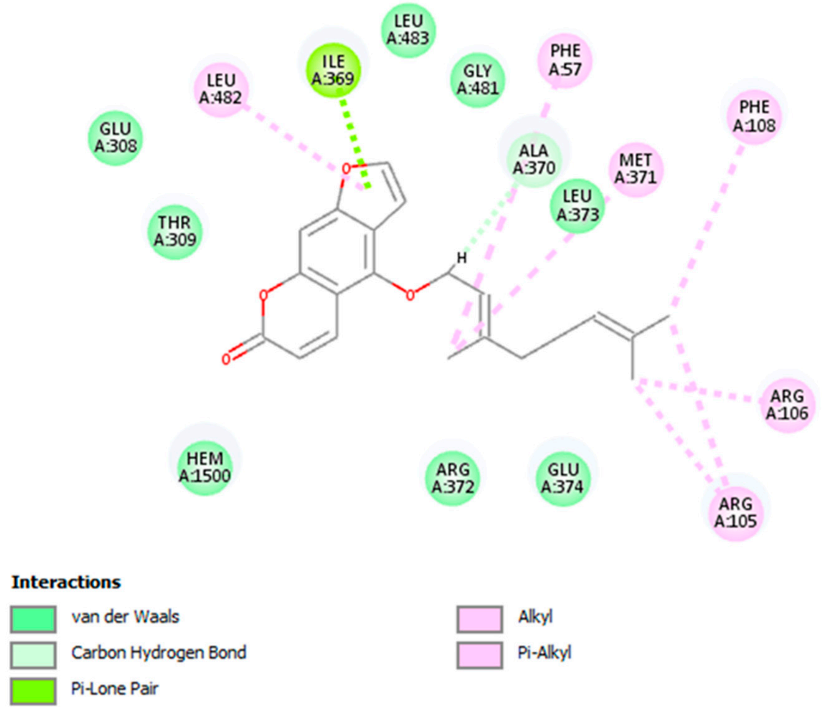

Figure S5. 2D interactions for bergamottin Pose 4

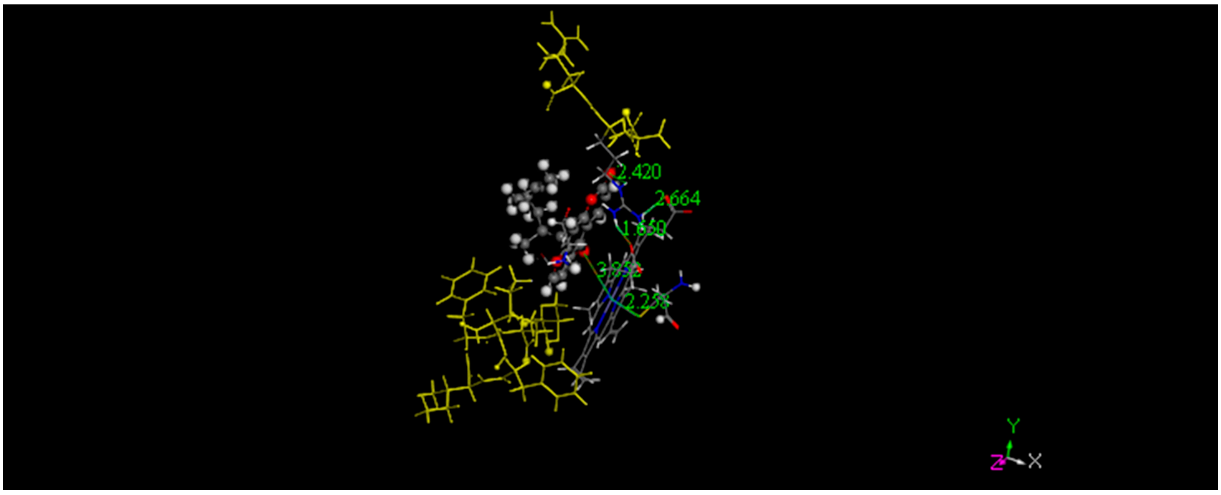

Figure S6. Atoms employed after docking procedure of pose 6, ready for DMol^3 minimization. Yellow atoms were kept fixed. Ball style terminal H atoms indicate polypeptide chain residue cuts. Basically, heme, bergamottin, the alkyl chain of Arg-105 and Ser-119 (except for the 2 H atoms substituting the polypeptide chain) atomic positions were allowed to refine.

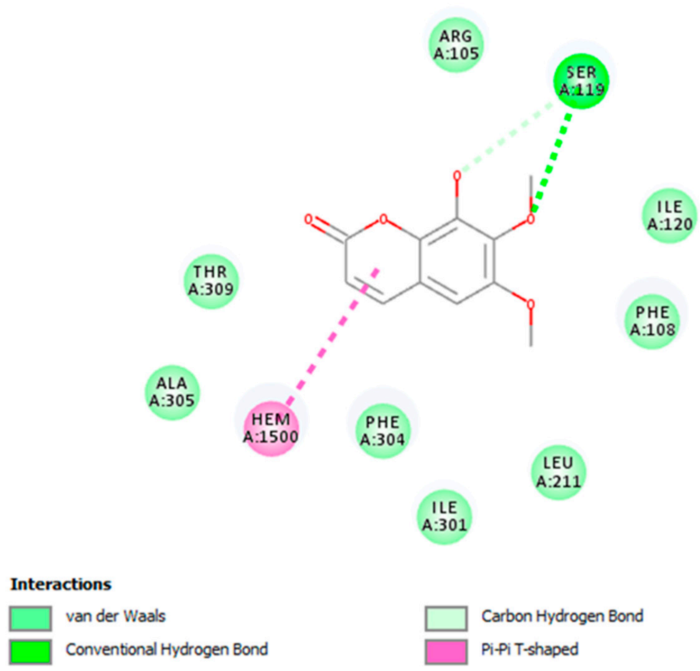

Figure S7. 2D diagram showing interaction of PDB 4D78 amino acid residues with fraxidin pose 8.

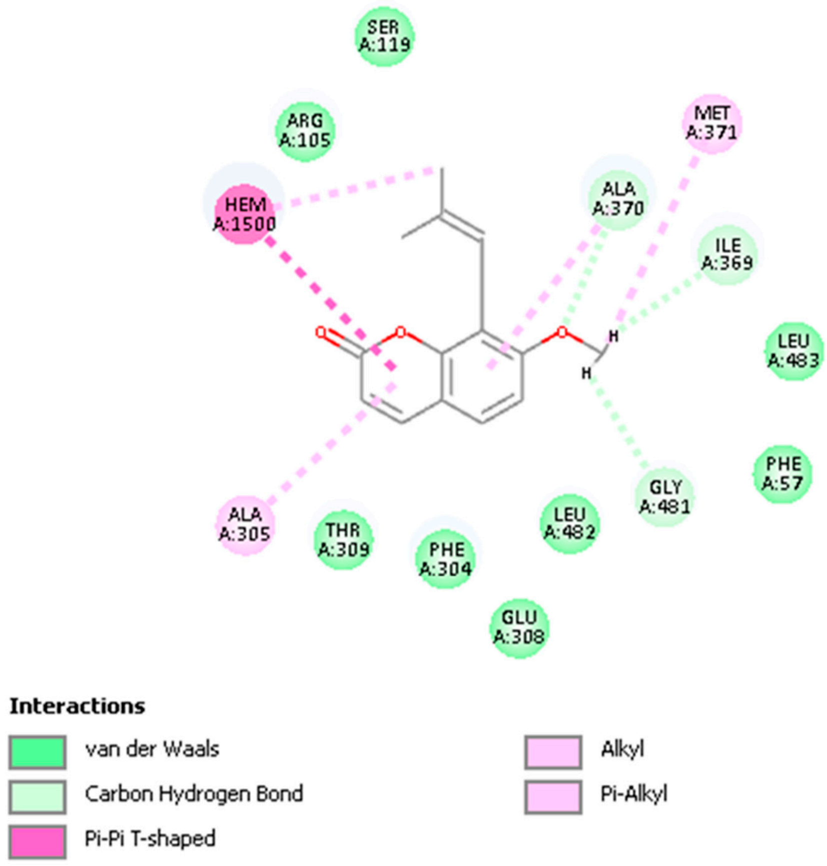

Figure S8. 2D display of residue interaction with Ost1, pose 1.

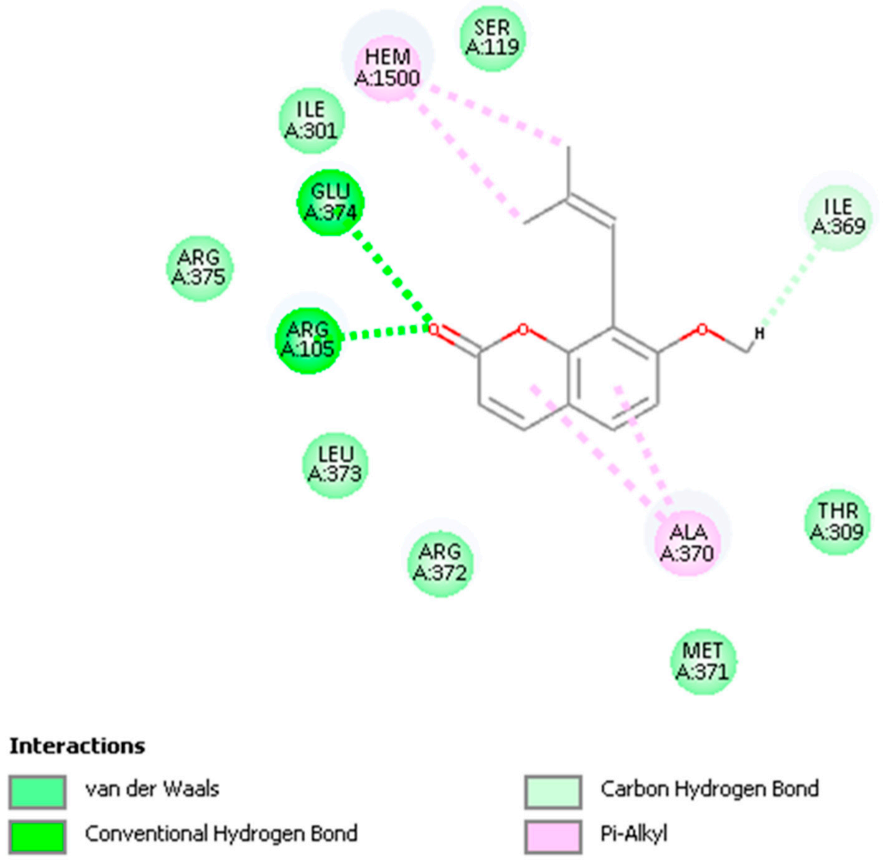

Figure S9. 2D display of residue interaction with Ost3, pose 3.

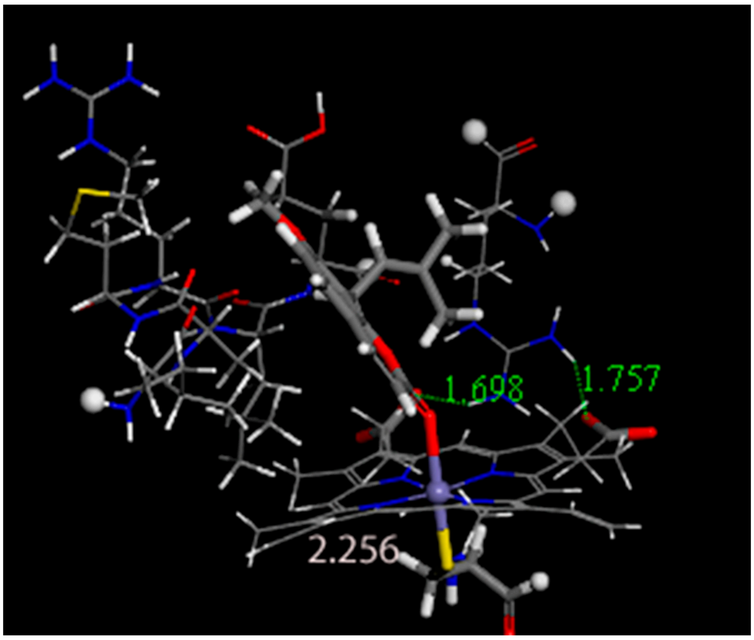

Figure S10. Docked Ost1 after DFT calculations. Distance Fe-O(carbonyl) 2.370 Å (not shown). Two hydrogen bonds between Arg105 and heme carboxylate 1.698 Å and 1.757 Å.
